# Web-Based Application for Reducing Methamphetamine Use Among Aboriginal and Torres Strait Islander People: Randomized Waitlist Controlled Trial

**DOI:** 10.2196/58341

**Published:** 2025-02-28

**Authors:** Rachel Reilly, Rebecca McKetin, Federica Barzi, Tayla Degan, Nadine Ezard, Katherine Conigrave, Julia Butt, Yvette Roe, Handan Wand, Brendan Quinn, Wade Longbottom, Carla Treloar, Adrian Dunlop, James Ward

**Affiliations:** 1 Wardliparingga Aboriginal Health Equity South Australian Health and Medical Research Institute Adelaide Australia; 2 School of Psychology University of Adelaide Adelaide Australia; 3 National Drug and Alcohol Research Centre University of New South Wales Sydney Australia; 4 Poche Centre for Indigenous Health University of Queensland Brisbane Australia; 5 School of Psychology Faculty of Arts University of Wollongong Wollongong Australia; 6 St Vincent's Hospital Sydney Australia; 7 Central Clinical School Sydney Medical School University of Sydney Sydney Australia; 8 Discipline of Psychology and Social Sciences School of Arts and Humanities Edith Cowan University Perth Australia; 9 Molly Wardaguga Institute for First Nations Birth Rights Charles Darwin University Casuarina, Northern Territory Australia; 10 The Kirby Institute University of New South Wales Sydney Australia; 11 Burnet Institute Melbourne Australia; 12 South Coast Aboriginal Medical Service Corporation Nowra Australia; 13 Centre for Social Research in Health University of New South Wales Sydney Australia; 14 School of Medicine and Public Health University of Newcastle Newcastle Australia

**Keywords:** methamphetamine, Aboriginal and Torres Strait Islander Health, web-based intervention, randomised controlled trial, therapeutic program, methamphetamine use, substance use, digital interventions, treatment, psychosocial wellbeing, effectiveness, app, psychosocial distress, mobile phone

## Abstract

**Background:**

Digital interventions can help to overcome barriers to care, including stigma, geographical distance, and a lack of culturally appropriate treatment options. “We Can Do This” is a web-based app that was designed with input from cultural advisors and end users to support Aboriginal and Torres Strait Islander people seeking to stop or reduce their use of methamphetamine and increase psychosocial well-being.

**Objective:**

This study aimed to evaluate the effectiveness of the “We Can Do This” web-based app as a psychosocial treatment for Aboriginal and Torres Strait Islander people who use methamphetamine.

**Methods:**

The web app was evaluated using a randomized waitlist controlled parallel group trial. Participants were Aboriginal and Torres Strait Islander people aged 16 years or older who self-identified as having used methamphetamine at least weekly for the past 3 months. Participants were randomized on a 1:1 ratio to receive either access to the web-based app for 6 weeks or a waitlist control group. Both groups received access to a website with harm minimization information. The primary outcome was days of methamphetamine use in the past 4 weeks assessed at 1, 2, and 3 months post randomization. Secondary outcomes included severity of methamphetamine dependence (Severity of Dependence Scale [SDS]), psychological distress (Kessler 10 [K10]), help-seeking behavior, and days spent out of role due to methamphetamine use.

**Results:**

Participants (N=210) were randomized to receive either access to the web-based app (n=115) or the waitlist control condition (n=95). Follow-up was 63% at 1 month, 57% at 2 months, and 54% at 3 months. There were no significant group differences in days of methamphetamine use in the past 4 weeks at 1 the month (mean difference 0.2 days, 95% CI –1.5 to –2), 2 months (mean difference 0.6 days, 95% CI –1 to 2.4 days) or 3 months (mean difference 1.4 days, 95% CI –0.3 to 3.3 days) follow-up. There were no significant group differences in K10 scores, SDS scores, days out of role, or help-seeking at any of the 3 follow-up timepoints. There was poor adherence to the web-based app, only 20% of participants in the intervention group returned to the web-based app after their initial log-in. Participants cited personal issues and forgetting about the web-based app as the most common reasons for nonadherence.

**Conclusions:**

We found poor engagement with this web-based app. The web-based app had no significant effects on methamphetamine use or psychosocial well-being. Poor adherence and low follow-up hindered our ability to accurately evaluate the effectiveness of the web-based app. Future web-based apps for this population need to consider methods to increase participant engagement.

**Trial Registration:**

Australian New Zealand Clinical Trials Registry ACTRN12619000134123p; https://www.anzctr.org.au/Trial/Registration/TrialReview.aspx?id=376088

**International Registered Report Identifier (IRRID):**

RR2-10.2196/14084

## Introduction

Methamphetamine use is an issue of deep concern in Australia, including in Aboriginal and Torres Strait Islander communities [1-4]. Although rates vary geographically and Aboriginal and Torres Strait Islander people tend to be underrepresented in national population surveys, the available data suggests that nationally, the rate of methamphetamine use is at least two times higher amongst Aboriginal and Torres Strait Islander people compared with the general population (2.9% vs 1.3%) [5,6]. Regular and frequent methamphetamine use is associated with long-term physical, psychological, and social harm [7,8]. Previous research has found that while both Aboriginal and Torres Strait Islander and non-Indigenous people who have recently used methamphetamine experienced harms such as an excess burden of psychological distress, exposure to violence, and economic exclusion [9], Aboriginal and Torres Strait Islander people who have recently used methamphetamine reported significantly higher rates of racial discrimination, excessive grief, perceived social isolation, and complex trauma than their non-Indigenous counterparts [1,9,10]. Key protective factors for Aboriginal and Torres Strait Islander participants included connection to culture and contact with elders [1].

These differences in the social processes leading to use, the contexts in which people use, and the community and cultural strengths that may assist recovery points to a need for culturally specific resources for Aboriginal and Torres Strait Islander people seeking to reduce or stop using methamphetamine [9]. In a context where demand for specialist alcohol and other drug (AOD) treatment across urban, rural, and remote settings in Australia is not met by available services, much of the burden for providing care to people who use methamphetamine falls to primary health care services [11]. The Novel Interventions to Address Methamphetamine in Aboriginal and Torres Strait Islander Communities (NIMAC) project was developed in response to calls from Aboriginal Community Controlled Health Services (ACCHS) and the community at large to find ways to better support ACCHS to reduce the harms associated with methamphetamine use [1,4,9].

Web-based interventions have emerged as a popular, feasible, and cost-effective way to provide treatment for substance use disorders. Evidence suggests that web-based interventions can be effective in the treatment of substance use disorders [12-14]. However, effectiveness can vary according to intervention type, characteristics of both the web-based app and end users, and substances targeted [12-15]. Although research in this space is ongoing, to date, there are limited interventions tailored to the treatment of methamphetamine use [12], and no known interventions targeting methamphetamine use for Aboriginal and Torres Strait Islander communities.

A scoping review of evidence on the uptake of web-based interventions in Indigenous communities internationally, found that such interventions can reduce barriers stemming from geographical distance, access to specialists, or stigma; they can support health practitioners provide better care to clients for a range of health conditions, but they need to be developed in partnership with communities [16]. There is evidence that web-based and digital interventions are acceptable and feasible in Aboriginal and Torres Strait Islander communities, with several such interventions developed for AOD assessment [17,18], psychosocial assessment and care planning [19], and suicide prevention [20]. Given the lack of specialist AOD services and the stigma associated with methamphetamine use, which is known to prevent help-seeking [4], a web-based intervention was considered a possible way to improve access to an evidence-based therapeutic intervention. Furthermore, in a survey conducted within the NIMAC project of 734 Aboriginal and Torres Strait Islander and non-Indigenous people who had used methamphetamine in the previous 12 months, we found that more than 90% of respondents had a mobile phone, and of these, 80% accessed the internet on their phone, which provided some initial indication that an online intervention may be feasible for this population [9].

The objective of this research was to test the effectiveness of the “We Can Do This” web-based app. The details of the web-based app have been reported elsewhere [21]. In brief, “We Can Do This” comprised 7 modules that were codesigned with clinicians, representatives of Aboriginal Community Controlled Health Services, and people with experience of use. Psychoeducational material, interactive exercises, planning, and goal-setting exercises were drawn from cognitive behavioral therapy (CBT), motivational interviewing, acceptance commitment therapy, and narrative approaches. Content was presented with films based on real narratives developed from qualitative interviews, and cultural content provided by advisors from diverse Aboriginal nations [21].

Our primary hypothesis was that participants who used the web-based app regularly for 6 weeks would have significantly reduced days of methamphetamine use in the last 4 weeks at 1, 2, and 3 months post intervention, compared with waitlisted participants with access to treatment as usual, including information about harm-minimization and where to access support. We also hypothesized that changes in secondary outcomes at 3-month follow-up would include increased help-seeking, decreased psychosocial distress, and fewer days out of role.

## Methods

### Trial Design

The web app was evaluated in a waitlist randomized controlled trial conducted in partnership with 10 urban and regional ACCHS across 6 Australian states and territories including South Australia (n=2), New South Wales (n=3), Queensland (n=1), Northern Territory (n=1), Western Australia (n=1), and Victoria (n=2). The protocol for this RCT study is already published [21]. The trial protocol was registered with the Australian and New Zealand Clinical Trials Registry (ANZCTRN12619000134123) on April 10, 2019 [21]. Participants were randomly allocated to the intervention or waitlist group with a 1:1 ratio. Random numbers were generated using the statistical software Stata (version 14.0; StataCorp) by a statistician not otherwise involved in the trial.

### Participants

Participants were eligible to participate if they were Aboriginal and Torres Strait Islander, aged 16 years or older, and self-identifying that they had been using methamphetamine about weekly for the previous 3 months. Eligibility was confirmed by participant self-report on entry to the web-based app platform. Participants were initially recruited through advertisements in health services and on social media. All participants were invited to provide informed consent through a web-based form before completing any study assessments.

Initially, the web app was designed to facilitate direct referral through the app to weekly support from health practitioners at partner health services. To enable wider recruitment, this function was modified to enable participants to self-refer to a local service regardless of geographical location within Australia. In consultation with the trial management committee, additional changes made posttrial commencement to aid recruitment were (1) providing a voucher for reimbursement for completion of the baseline survey (Aus $25), (2) increasing the value of voucher reimbursement for the first follow-up survey (from Aus $20 to Aus $25), (3) introducing peer recruitment, where peers were offered Aus $20 for every participant referred to the project, (4) reducing the number of questions in the assessments to reduce drop out before reaching randomization, and (5) extending the recruitment period. During this period the average conversion rate was approximately Aus $1=US $0.7.

### Procedures

Advertisements directed participants to a web landing page that included information about the project in both written and audio-visual format. After providing consent, all participants completed a survey at baseline before randomization. After participants completed the baseline survey, they were randomized to either the intervention group or a waitlist control group according to a 1:1 ratio. The randomization schedule was developed by a statistician who was not involved with study participants (HW). Random numbers were generated using the statistical software Stata. All participants were followed up at 1, 2, and 3 months post baseline. Assessments were completed through self-reporting online, with an option to have a researcher call to complete the assessment over the phone if this was considered easier. Attempts were made to contact all participants by email, SMS text messages, and phone for follow-up assessments if they failed to respond to follow-up assessments within 2 weeks. After 120 days post baseline, participants were considered lost to follow-up.

### Intervention

The intervention group received access to the “We Can Do This” web app for 6 weeks. Participants could access the 7 modules on smartphones, tablets, or computers, and complete them in a self-paced manner. There was no restriction on the order in which participants could complete the modules. To commence the intervention, participants set up an account in the web app. During the 6 weeks of access to the intervention, they received weekly reminders to log in by email, with encouragement to log in for around half an hour per week. If the system identified that a participant had not logged in for 7 days, this triggered a text message with a prompt to log in and a link to the web app [21].

### Control Group

Participants allocated to the waitlist group were directed to the study website, with links to harm-minimization information and options for self-referral to AOD counseling. Participants in this group were sent a link with access to the web app immediately following the completion of their final follow-up survey. The waitlist control group then had access to the web app for 6 weeks.

### Measures

#### Primary Outcome

The primary outcome was the number of days the participant used methamphetamine in the past 4 weeks, assessed at baseline and 1, 2, and 3 months post baseline using the Australian Treatment Outcome Profile (ATOP [22]).

#### Secondary Outcomes

Secondary outcomes were the help-seeking intention, assessed using the General Help-Seeking Questionnaire [23], readiness to change (Readiness to Change Questionnaire [24]), psychological distress (Kessler 10 [25]), severity of dependence on methamphetamine in the past month (Severity of Dependence Scale (SDS) [26]), and other substance use during the past month, including cannabis, alcohol, tobacco, benzodiazepines, heroin, other opioids, cocaine, ecstasy, and other drugs (ATOP [22]), and days out of role during the past month, resulting from methamphetamine use [27].

#### Other Measures

Usability and acceptability of the web-based app were measured using the Internet Intervention Adherence Questionnaire [28], which includes questions about barriers and facilitators to using the program and optional free-text to report any criticisms or other feedback.

The overall feasibility of the web-based intervention was assessed by observing the uptake and use of the web-based intervention (number of modules accessed). The number of visits to the web-based app overall and per module was monitored using Google Analytics. The Internet Intervention Adherence Questionnaire [28] was included in the second follow-up questionnaire for the intervention group.

#### Statistical Methods

Based on our previous research, we expected a baseline mean of 16 days of methamphetamine use in the past 4 weeks [29,30]. Our original sample size of 288 participants was intended to detect a reduction from a mean of 16 (SD 14.5) days of use in the past 4 weeks to a mean of 8 use days in the past 4 weeks at each of the follow-up points, with 90% power at *P*<.01, allowing for 30% attrition (ie, 100 per group at follow-up). Our final sample included 210 participants, and we observed a 40% attrition. With this sample, we were able to detect a reduction from 16 days of use in the past 4 weeks to a mean of 6 use days in the past 4 weeks at each of the follow-up points, with 90% power at *P*<.01.

#### Data Analysis

An intention-to-treat analysis was used to compare the primary and secondary outcomes for the intervention versus the waitlist, 2 and 3 months from baseline. Descriptive statistics of baseline characteristics were provided by the randomization group. Continuous variables were summarized with mean and SD when appropriate, or median and IQR when not normally distributed, and categorical variables were summarized with frequency and percentage.

Multilevel mixed-effects generalized linear model, with either a Gaussian, Poisson, or robust Poisson distribution as appropriate for each specific outcome, were used to estimate change between (1) baseline and 1-month assessment, (2) baseline and 2-month assessment, and (3) 3-month assessment. To compare the changes described above between study arms, the models included a categorical variable for the study arms, a categorical variable for each time point, and an interaction term between arm and time. The use of mixed models has the advantage of allowing the modeling of the correlation between repeats of outcomes recorded within the same participant. A per-protocol analysis was carried out to assess the effect of the intervention for those participants who accessed the app at least once versus those participants who never accessed the app. Sensitivity analyses were also carried out that included baseline outcomes in the models when descriptive analyses showed important between intervention arm differences for baseline scores.

Statistical significance was set at *P*<.01. Statistical analysis was conducted using Stata (version 18) statistical software.

### Ethical Considerations

The trial was approved by ethics committees in all relevant jurisdictions: Aboriginal Health Research Ethics Committee of South Australia (04-19-810), Aboriginal Health and Medical Research Council of New South Wales (1484/19), University of Queensland (2020001710), Menzies School of Health Research (2019-3330), Central Australian Human Research Ethics Committee (CA-20-3779), Western Australian Aboriginal Health Ethics Committee (1002), St Vincent’s Hospital Melbourne Human Research Ethics Committee (52756: 149/20), and University of Tasmania (23229).

## Results

**Figure 1:**
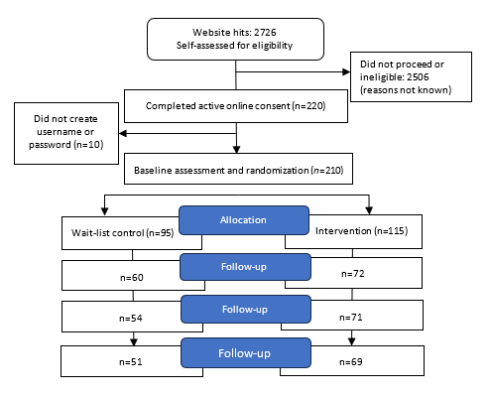
CONSORT flow diagram: Numbers of participants who self-assessed for eligibility, completed online consent and baseline assessment, were randomized to intervention or wait-list control, and retained for follow-up at 1, 2, and 3 months.

Recruitment continued for a total of 22 months from December 2019 to October 2022 with the final follow-up completed in February 2022. Out of the 210 participants who completed the baseline survey, 95 were randomized into the waitlist control group and 115 into the intervention group. Participant flow and attrition are shown in Figure 1 (Multimedia Appendix 1 shows the CONSORT [Consolidated Standards of Reporting Trials] checklist).

Baseline participant characteristics are shown in Table 1. In both groups, close to 90% were Aboriginal, with the remainder either Aboriginal and Torres Strait Islander or Torres Strait Islander. Participants’ mean age was 33.9 (SD 8.7) years. They had been using methamphetamine from a median age of 21 (IQR 18-28) years and had used methamphetamine on a median of 20 (IQR 13-25) days in the past 4 weeks. A total of 82 % smoked tobacco (92% of them, daily). Other substance use consisted mainly of cannabis (65%) and alcohol (60%), with cannabis being used more often than alcohol (median 13, IQR 0-28 days for cannabis vs 11, IQR 1-28 days for alcohol). Other drug use was less common (Table 1).

There were more men in the intervention group (64%) compared with the control group (43%), but the 2 groups were broadly equivalent on other sociodemographic variables and drug use (Table 1).

**Table 1:** Summary of baseline socioeconomic factors and drug use by study arm and overall.

		Intervention (n=115)	Waitlist (n=95)	Total (N=210)
Age (n=208), mean (SD)		34.5 (9.2)	33.2 (8.1)	33.9 (8.7)
**Gender (n=209)**				
	Male, n (%)	49 (43)	60 (63.2)	109 (52.2)
**Sexual identity (n=210)**				
	LGBTQI^a^, n (%)	9 (7.8)	19 (20)	28 (13.3)
**Aboriginality (n=206)**				
	Aboriginal, n (%)	100 (89.3)	85 (90.4)	185 (89.8)
**Relationship status (n=210)**				
	Single, n (%)	70 (60.9)	55 (57.9)	125 (59.5)
**Employment status (n=210)**				
	Working (at all), n (%)	24 (20.9)	35 (36.8)	59 (28.1)
**Highest level of education (n=208)**				
	Completed high school, n (%)	42 (36.8)	36 (38.3)	78 (37.5)
	Age at first meth use (years), median (IQR)	22 (19-29)	21 (18-27)	21 (18-28)
	Days of methamphetamine use in the past 4 weeks, median (IQR)	19 (14-25)	20 (11-27)	20 (13-25)
**Route of methamphetamine use (n=209), n (%)**				
	Injecting	53 (46.1)	44 (46.8)	97 (46.4)
	Smoking	61 (53)	46 (48.9)	107 (51.2)
	Other	1 (1)	4 (4.3)	5 (2.4)
**Other drug use in the past 4 weeks, n (%)**				
	Alcohol	71 (61.7)	56 (59)	127 (60.3)
	Cannabis	57 (60)	57 (60)	114 (60)
	Tobacco	93 (81.6)	78 (82.1)	171 (81.8)
	Cocaine	14 (12.2)	14(14.7)	28 (13.5)
	Heroin	12 (10.4)	6 (6.3)	18 (8.6)
	Other opioids	20 (17.5)	18 (20)	38 (18.2)
	Benzodiazepines	40 (34.8)	31 (33.3)	71 (34.1)
	Other drugs	13 (11.3)	6 (6.3)	19 (9)

^a^LGBTQI: lesbian, gay, bisexual, transgender, queer/questioning, or intersex.

### Outcomes

**Figure 2:**
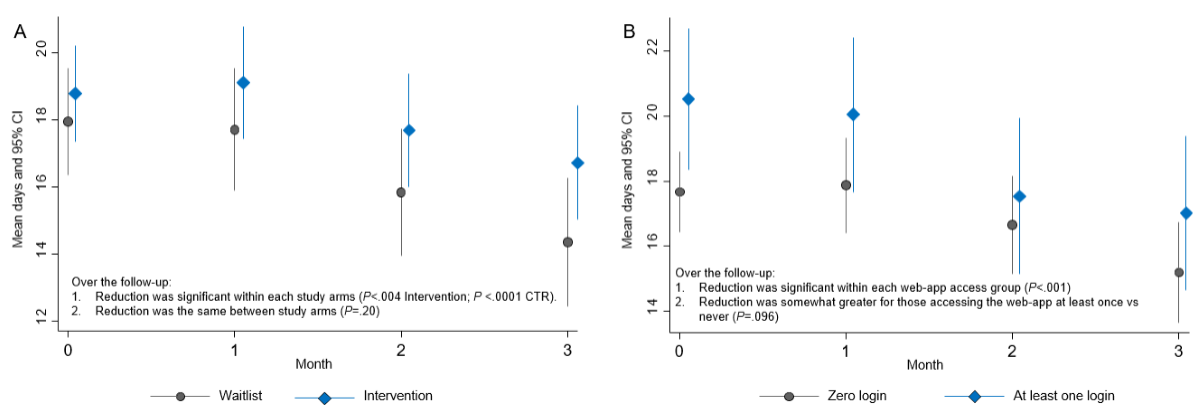
A: Mean days and 95% CI of methamphetamine use at baseline (month 0) and at each of the 3 follow-up months—intervention arm versus waitlist arm. B: Mean and 95% CI days of methamphetamine use at baseline (time 0) and at each of the 3 follow-up months for the intervention arm only, for participants who logged in the “We Can Do This” web app at least once after initial sign up versus those who never logged-in the web app after initial sign up.

Days of methamphetamine use reduced significantly in both intervention and control groups (*P*<.04), but there was no significant difference between the groups at 1, 2, or 3 months post baseline (Figure 2A). As shown in Table 2, there were no significant group differences in the secondary outcomes, including psychological distress (K10), the severity of dependence, help-seeking, days out of role, and other drug use.

Usage data acquired through Google Analytics showed a large proportion (80%) of participants logged into the web app once and did not subsequently return. Retrospective analysis of the intervention condition only, comparing those participants who did not log into the web app again after initial sign-up (“never”) versus those who logged in at least once more (“at least once”), showed a significant reduction in use amongst those who returned (Figure 2B).

Estimates were derived using multilevel mixed-effects generalized linear models, including random effects for the intercept and for time, with either a Gaussian, Poisson, or robust Poisson distribution as appropriate for each specific outcome. The treatment effect is calculated as the difference between the intervention and waitlist at 1-month assessment, 2-month assessment, and 3-month month assessment. The use of multilevel mixed effect models allows for estimates of treatment effects that are adjusted for between study arms differences at baseline.

**Table 2:** Mean (95% CI) and rate (95% CI) for each study outcome at baseline, month 1, month 2, and month 3 in each study arm, and corresponding treatment effect (difference of means, relative rate, and 95% CI).

				Baseline		Month 1		Month 2		Month 3
**Primary outcome**										
	**Days of methamphetamine use—last 4 weeks, mean (95% CI)**									
		Intervention	19.5 (17.3-21.8)		19.1 (16.7-21.4)		17.8 (15.6-20)		17.2 (15-19.3)	
		Waitlist	18.5 (16.4-20.8)		17.8 (15.4-20.2)		16.1 (13.9-18.3)		14.8 (12.7-16.9)	
		Treatment effect	—^a^		0.2 (–1.5 to 2.0)		0.6 (–1.0 to 2.4)		1.4 (–0.3 to 3.3)	
**Secondary outcomes**										
	**K10** ^b^ **score** **, mean (95% CI)**									
		Intervention	31.3 (29.6-33)		31.7 (29.5-33.8)		29.2 (27.1-31.2)		28.2 (26.2-30.3)	
		Waitlist	29.8 (27.9-31.6)		28.7 (26.4-31)		26.6 (24.3-28.9)		25.9 (23.6-28.2)	
		Treatment effect	—		1.4 (–1.9 to 4.7)		1 (–2.2 to 4.2)		0.8 (–2.4 to 4.1)	
	**SDS** ^c^ **score** **, mean (95% CI)**									
		Intervention	8.7 (8.1-9.3)		8.4 (7.7-9.1)		8.7 (8.0-9.4)		8.5 (7.8-9.3)	
		Waitlist	8.5 (7.8-9.1)		8.7 (8-9.5)		7.9 (7.1-8.7)		7.4 (6.7-8.3)	
		Treatment effect	—		–0.5 (–1.6 to 0.5)		0.6 (–0.5 to 1.7)		0.9 (–0.2 to 2)	
	**Days out of role** **rate (95% CI) of 0 to 1 day a month**									
		Intervention	26.3 (18.1-34.4)		26.4 (16.3-36.6)		14.1 (6.1-22.1)		16.3 (7.6-25)	
		Waitlist	27.3 (18.2-36.4)		29.1 (17.5-40.6)		30.2 (17.9-42.4)		31.1 (18.3-43.7)	
		Treatment effect, RR^d^ (95% CI)	—		0.94 (0.48-1.8)		0.48 (0.22-1.07)		0.55 (0.26-1.13)	
	**Days cut back on role** **rate (95% CI) of 0 to 1 day a month**									
		Intervention	33.3 (24.7-42)		33.3 (22.1-44.4)		29 (18.3-39.7)		25.4 (14.9-35.8)	
		Waitlist	27.3 (16.2-38.4)		29.1 (14.7-43.4)		30.2 (14.8-45.5)		31.1 (15.3-46.9)	
		Treatment effect, RR (95% CI)	—		0.94 (0.4-2.21)		0.48 (0.19-1.26)		0.55 (0.21-1.40)	
	**Sought help** **(Yes), rate (95% CI)**									
		Intervention	21 (10.6-21.5)		22.9 (9.3-36.5)		22.4 (9.1-35.7)		28.3 (12.5-44.0)	
		Waitlist	20 (9.4-30.7)		8.7 (0.1-16.8)		12.9 (2.3-23.4)		20.7 (6.4-34.9)	
		Treatment effect, RR (95% CI)	—		2.52 (0.75,8.4)		1.66 (0.54-5.06)		1.30 (0.48-3.55)	
	**Days of alcohol use** **, mean (95% CI)**									
		Intervention	13.1 (10.6-15.5)		11.3 (9.4-13.2)		10.1 (8.5-11.7)		8.6 (6.9-10.3)	
		Waitlist	13.3 (10.6-16.0)		10.5 (8.6-12.4)		8.0 (6.5-9.5)		8.0 (6.1-9.9)	
		Treatment effect days	—		1.0 (–1.6 to 3.3)		2.3 (–0.3 to 4.4)		0.8 (–2.7 to 3.9)	
	**Cannabis use,** **rate (95% CI)**									
		Intervention	77.4 (12.6-98.2)		56.9 (9.1-100)		57.5 (9.2-100)		54.5 (8.7-100)	
		Waitlist	53.3 (8.4-98.2)		36.2 (5.5-66.8)		43.9 (6.7-81.2)		38.1 (5.8-70.4)	
		Treatment effect, RR (95% CI)	—		1.08 (0.93-1.25)		0.90 (0.85-1.15)		0.98 (0.85-1.14)	
	**Tobacco use** **(any use), rate (95% CI)**									
		Intervention	81.1 (73.6-88.6)		85.8 (77.8-93.9)		85.5 (77.2-93.8)		78.6 (69-88.3)	
		Waitlist	81.4 (73.1-81.4)		70.8 (58.8-82.7)		75.6 (86.9-64.2)		71.2 (83.4-59.1)	
		Treatment effect, RR (95% CI)	—		2.1 (1.01-4.1)		1.7 (0.81-3.61)		1.37 (0.7-2.66)	
	**Any other drug use** ^e^ **,** **rate (95% CI)**									
		Intervention	47.8 (38.6-57)		35.9 (24.8-47.0)		41.2 (29.6-52.8)		38.7 (27.1-50.3)	
		Waitlist	46.3 (36.1-56.4)		48.1 (35.4-60.8)		46.9 (33.5-60.4)		43.5 (30-56.9)	
		Treatment effect, RR (95% CI)	—		0.73 (0.47-1.11)		0.85 (0.56-1.29)		0.86 (0.56-1.34)	

^a^Not available.

^b^K10: Kessler 10.

^c^SDS: Severity of Dependence Scale.

^d^RR: risk ratio.

^e^Any use of heroin, benzodiazepine, heroin, other opioids, cocaine, ecstasy, or other drugs.

### Usability and Adherence

The most common issues participants cited as obstacles to completing the web app (either a “little problem” or a “major problem”) were “personal issues stopped me from using the web program” (55%) and “I just forgot to go to the web program” (51%). Problems with the internet or computer were cited by 39%-41% of participants. Issues with navigability and presentation of the web app were less often cited as barriers (20%-29%), although 41% said the program was not useful and 43% said they simply did not want to do it. Practical barriers, like other people using the computer or not having enough time to do the intervention, were cited by 44% and 42% respectively. Items that were most frequently considered major problems for using the web app (>10%) were forgetting to use it, having personal issues, believing it would take too long, not having time, and the computer not working or having problems.

The majority of respondents indicated that several matters were not problems that prevented them from using the web app, including internet connectivity, comprehension of the program, navigation, work issues getting in the way, difficulty reading the screen, or too many words. The frequency of responses to all usability and adherence questions is included in Table S1 in Multimedia Appendix 2.

## Discussion

### Principal Findings

We found that the web app, “We Can Do This,” which targeted Aboriginal and Torres Strait Islander people, was not effective in reducing methamphetamine use. This is consistent with outcomes of a similar but nontargeted internet-based intervention for stimulant use [31,32], which did not produce significant changes in stimulant use. However, it is inconsistent with the broader evidence base for internet-based interventions, which have been found to produce significant, if modest, reductions in substance use [12,33]. These include effective CBT-based online interventions for methamphetamine use [34]. The web app did not produce expected increases in help-seeking intention, motivation to reduce methamphetamine use, and reductions in days out of role, benefits that have been found with nontargeted internet interventions for stimulant use [31,32].

The most likely explanation for the failure to yield expected benefits from “We Can Do This” is poor engagement with this self-directed web-based intervention, with only a small proportion of participants completing the web app modules. This interpretation of the results is supported by our per-protocol analysis, which found evidence of a reduction in methamphetamine use amongst participants who completed at least one module of the intervention. This could suggest that the web app has the potential to be effective for those people who return to it for two or more sessions. However, delivered in its current form, it is unlikely to result in any population shift in methamphetamine use. Keeping people engaged with health-related websites and apps is a universal problem [35,36] and it is also a specific challenge in trials of psychosocial interventions targeting stimulant use [37].

In our case, simply forgetting to use the web app was one of the main reasons cited as an obstacle to engagement. Retention and engagement strategies used in this study included SMS text messages and email reminders. However, more aggressive tactics, such as push notifications, in-app messages, and “gamification” mechanics (using rewards, competition, “unlocking” levels, or time-limited challenges) could be used to increase retention and engagement [15,38]. Contingency management can also be used to increase treatment attendance and engagement with treatment-related activities [39] and may be one option to increase engagement with the web app. Significant work would be required to update the web app to incorporate more of these features for it to be successfully offered as a stand-alone, self-directed intervention available in the community.

Other barriers to not completing the web app were mostly external (eg, personal issues, not finding time, and computer problems) rather than barriers related to the content or layout of the web app. Importantly, personal issues were a major barrier. Further investigation of the type of personal issues that impeded using the web app would be needed to understand the implications of this finding. However, it may reflect the complex and challenging lifestyle associated with having a stimulant use disorder. It may also reflect the mood disturbances commonly experienced by people with a stimulant use disorder, both related to co-occurring mental disorders, but also symptoms of withdrawal from methamphetamine. Further qualitative research and codesign of web apps are likely to be needed to understand and overcome these barriers to adherence.

There are also factors related to the target population that have been associated with poor retention and engagement. At a population level, life stress is generally higher for Aboriginal people in Australia compared with non-Indigenous people [40]; low educational attainment and a lack of income increase the risk of dropping out from alcohol and other drug treatment programs [41], and frequent substance use is associated with poor treatment retention [42,43]. Cognitive factors, although inconsistently associated with treatment outcomes in other settings [42], may be relevant to a program, such as “We Can Do This,” which requires both self-motivation and concentration. In light of this, there may be value in exploring alternative approaches to delivery, for example, clinician-assisted web apps, single brief interventions, or web apps that focus on a broader range of drug use and treatment scenarios. Qualitative research with people who have attempted to engage with the web app will provide a deeper understanding of these issues.

### Limitations

The study was subject to several limitations. The major limitation of this study was poor adherence to the internet intervention, which limited our ability to detect a treatment effect in the intention-to-treat analysis. This clearly has implications for any conclusions that can be drawn about the useability of the web app in its current form. Dropout from the assessments may also have biased findings, particularly in the treatment completers analysis. Data on drug use relied on self-report and could not be verified. However, self-reporting in research studies shows high concordance with biological indicators of drug use [44].

The anonymous data collection was deemed necessary to enable participation from people who may otherwise not disclose information about their drug use, but it did limit the ability to confirm the veracity of the data. Although we carefully monitored the use of the web app to identify any instances of unusual access (such as multiple sign-ups from 1 IP address) and contacted several participants by phone to complete assessments (as per protocol), we have no way of being certain that all participants met the eligibility criteria.

### Conclusion

We did not demonstrate the effectiveness of this web app in reducing methamphetamine use or increasing psychosocial well-being. Overall, engagement with the web app was poor and hindered our ability to evaluate its effectiveness. Future web apps for this population need to consider methods to increase participant engagement. Understanding the unique challenges and strengths of Aboriginal and Torres Strait Islander participants and communities, and ongoing guidance from communities and ACCHSs will be key to ongoing efforts to identify accessible and effective interventions for methamphetamine use.

## Appendix

Multimedia Appendix 1 CONSORT checklist.

Multimedia Appendix 2 Questions on useability and adherence, intervention group only, and second follow-up.

## Abbreviations

ACCHS: Aboriginal Community Controlled Health Services
AOD: alcohol and other drug
ATOP: Australian Treatment Outcome Profile
CBT: cognitive behavioral therapy
CONSORT: Consolidated Standards of Reporting Trials
K10: Kessler 10
NIMAC: Novel Interventions to Address Methamphetamine in Aboriginal and Torres Strait Islander Communities
SDS: Severity of Dependence Scale

## Data Availability

The datasets generated during and/or analyzed during this study are not publicly available due to the sensitivity of the data for Aboriginal and Torres Strait Islander people, and in line with ethical and data sovereignty principles.
