# Supplementary material for: Web-Based Application for Reducing Methamphetamine Use Among Aboriginal and Torres Strait Islander People: Randomized Waitlist Controlled Trial
Source: J Med Internet Res. 2025 Feb 28;27:e58341. doi: 10.2196/58341 (PMC11909485; doi:10.2196/58341)
Supplement: Multimedia Appendix 2 [file jmir_v27i1e58341_app2.docx]

**Multimedia Appendix 1**

**Table S1: Questions on useability and adherence, intervention group only, second follow-up.**

| **Item** | **N** | **Not a problem n(%)** | **Little problem n(%)** | **Major problem n(%)** |  |
| --- | --- | --- | --- | --- | --- |
| My internet connection did not work | 73 | 37(51) | 23(32) | 5(7) |  |
| My connection was too slow | 72 | 40(56) | 18(35) | 4(6) |  |
| Computer not working or having problems | 71 | 33(46) | 18(25) | 10(14) |  |
| Web program did not seem useful | 73 | 32(44) | 21(29) | 9(12) |  |
| Web program too hard to understand | 71 | 42(59) | 12(17) | 2(3) |  |
| Web program too hard to navigate | 71 | 39(55) | 18(26) | 2(3) |  |
| Work issues stopped me from using it | 68 | 36(53) | 13(21) | 6(9) |  |
| Personal issues stopped me from using it | 74 | 19(26) | 29(39) | 12(16) |  |
| Other people using the computer | 70 | 26(37) | 22(31) | 9(13) |  |
| I didn’t have time to go to the program | 74 | 32(45) | 21(29) | 10(13) |  |
| I just forgot | 73 | 26(36) | 23(32) | 14(19) |  |
| I thought the program was going to take too long | 70 | 29(41) | 20(29) | 8(11) |  |
| The web program had too many words | 70 | 36(51) | 14(20) | 4(6) |  |
| The screen was hard to read | 70 | 38(54) | 11(16) | 6(9) |  |
